# Co-circulation of all the four dengue virus serotypes and detection of a novel clade of DENV-4 (genotype I) virus in Pune, India during 2016 season

**DOI:** 10.1371/journal.pone.0192672

**Published:** 2018-02-22

**Authors:** Shubham Shrivastava, Divya Tiraki, Arundhati Diwan, Sanjay K. Lalwani, Meera Modak, Akhilesh Chandra Mishra, Vidya A. Arankalle

**Affiliations:** 1 Department of Communicable Diseases, Interactive Research School for Health Affairs, Bharati Vidyapeeth Deemed University, Pune, Maharashtra, India; 2 Department of Medicine, Bharati Vidyapeeth Deemed University Medical College, Pune, Maharashtra, India; 3 Department of Pediatrics, Bharati Vidyapeeth Deemed University Medical College, Pune, Maharashtra, India; 4 Department of Microbiology, Bharati Vidyapeeth Deemed University Medical College, Pune, Maharashtra, India; Emory University School of Medicine, UNITED STATES

## Abstract

Dengue is the most common mosquito-borne viral infection in tropical and sub-tropical countries. In recent years, India has reported increased incidences of concurrent infection with multiple serotypes of dengue viruses (DENV). In the present study, we have characterized DENV circulating during a single season of 2016 in Pune, India. A total of 64 serum samples from NS1 ELISA positive dengue patients were used for PCR amplification of CprM region of the viral genome and sequencing. Phylogenetic analysis documented circulation of all the four DENV serotypes with predominance of DENV-2 (40.6%). DENV genotyping classified DENV-1 to Genotype V, DENV-2 to Genotype IV, DENV-3 to Genotype III and DENV-4 to Genotype I. Further analysis revealed emergence of a novel clade (D) of genotype I of DENV-4. Subsequent isolation of three DENV-4 viruses in cell culture followed by complete genome sequence analysis confirmed this observation. Additionally, a new genotype within serotype-4 with >6.7% sequence variation from other genotypes was identified. This first report of significant co-circulation of all the four serotypes in a single outbreak in Pune reconfirms need for molecular monitoring of DENV.

## Introduction

An estimated 40% of the global population (~3.9 billion) is at risk of dengue virus (DENV) infection [1, 2]. About 2.5% of people affected with severe dengue die each year [3]. The disease is endemic in more than 125 countries and the spread to newer areas is mainly attributed to returning travelers from endemic countries [4, 5]. There are four serotypes of DENV (DENV-1 to -4) and all of them can cause dengue fever (DF), a self-limiting febrile illness. A variable proportion of patients progress to life threatening dengue hemorrhagic fever (DHF) characterized by thrombocytopenia and hemorrhage, and dengue shock syndrome (DSS) due to excessive plasma leakage [6, 7].DENV has been in circulation in the Indian subcontinent since 1950s [8]. The first virologically proven epidemic of DF occurred in Kolkata in 1963–1964 and at present the virus has spread to 35 states and union territories in the country (NVBDCP, http://nvbdcp.gov.in/den-cd.html) [9].

On account of sequence variability, dengue serotypes are further classified into distinct genotypes that differ >6% within a single serotype [10–12].Emergence of new serotype or lineage \ clade shifts in circulating DENV genotypes led to enhanced severity during dengue outbreaks [13–17]. A lineage shift in DENV-3 was reported to cause severe disease in Sri Lanka [13, 18]. Emergence of genotype III of DENV-3in 2005 resulted in dengue outbreak in Northern India [19]. Recently, emergence of Asian or genotype I of DENV-1 also caused large outbreak of dengue with 12,000 cases in Tamil Nadu, South India [20].

All the four serotypes of DENV have circulated in India at different times, but generally one serotype dominates a given outbreak. Dengue outbreak in 1996 in Delhi was caused by genotype IV of DENV-2 replacing genotype V isolates of 1957 and 1967[21]and virus remained in circulation till 2002. Second outbreak in 2003 in Delhi was due to emergence of DENV-3 which remained as dominant serotype till 2006 [19]. Over a period from 2007–2009, DENV-1 became the predominant serotype in Delhi by replacing DENV-2 and DENV-3 [22]. Earlier dengue outbreaks were attributed to sudden emergence of serotype or genotype that co-circulate along with existing genotype for some time before getting replaced by others in subsequent years. In recent years, co-circulation of multiple serotypes has been reported from different parts of India [23]. High percentage of co-infection with more than one serotype was also observed with increased disease severity [24–26]. In 2017, co-circulation of all four DENV serotypes in single outbreak was reported from Odisha [27] and Hyderabad [28].

Pune city, western India with a population of 112 million (census 2011) is endemic for dengue [29]. In view of the possibility of introduction of dengue vaccine in near future, it is essential to understand the type and proportion of circulating DENV strains. The present study reports molecular characterization of dengue viruses circulating in Pune during the 2016-dengue season.

## Methods

### Sample collection

Patients presenting with dengue like symptoms for < 4days to the Medicine and Pediatric OPDs of the Bharati hospital, a tertiary care hospital from Pune were included in the study. To avoid second prick, consent for the use of blood sample for dengue molecular studies was obtained from all the suspected patients. This included written informed consent from the parents (subjects below 7 years of age), written informed assent and consent (subjects and their parents respectively, age group 7–17 years) and written informed consent (subjects above 17 years of age). NS1 positive (Dengue Early ELISA, Panbio, Windsor, Qld, Australia), leftover serum samples (n = 120) were collected from the diagnostic laboratory of the hospital. The study was approved by Institutional Ethics Committee, Bharati Vidyapeeth Deemed University, Pune with approval number IEC/2017/04.

### Virus isolation

One day prior to infection, 1 x 10^4^ Vero cells were seeded in each well of 96-well plate and incubated at 37°C in 5% CO_2_ incubator. 100μl of 10-fold serially diluted patient’s serum in quadruplate wells in 96-well plate was used to infect Vero cells grown in Minimum Essential Medium (MEM, Gibco, Thermo Scientific) containing 2% fetal bovine serum, 1% penicillin and streptomycin. 7 days post infection, culture supernatant was harvested from NS1 positive wells and aliquots were stored at -80°C.

### Viral RNA extraction

Total RNA was extracted from 140μl of human serum or cell culture isolates using a QIAmp viral RNA kit (QIAGEN, INC, Valencia, CA), as per manufacturer’s protocol. RNA was eluted in 50μl of AVE buffer provided with the kit. For a conventional gel-based PCR, a minimum of one negative control every four samples with no presence of target RNA was included as a part of the extraction procedure.

### cDNA synthesis

Dengue specific viral RNA was reverse transcribed and amplified for CprM region of the viral genome as reported by Chien et al (2006) [30]. For this, single-stranded cDNA was synthesized from total RNA using the high capacity cDNA reverse transcription kit (Invitrogen). Briefly, 10μl of the extracted RNA was added to the 2 X RT master mix consisting of 2μl of 10X RT buffer, 0.8μl of 100mM dNTP mix, 2μl of reverse primer D2 (TTGCACCAACAGTCAATGTCTTCAGGTTC-616) and 1μl of MultiScribe reverse transcriptase. The reaction was then subjected to reverse transcription at 25°C for 1min, 37°C for 120min, 85°C for 5min. The prepared cDNA was immediately used or stored at -20°C until use.

### PCR and sequencing

CprM region was PCR amplified using AmpliTaq polymerase kit (Invitrogen). 5μl of the synthesized cDNA was then added to the PCR mix containing 10μl of PCR buffer, 10μl of MgCl_2_, 5μl of primers mD1 (134-TCAATATGCTGAAACGCGAGAGAAACCG) and D2 each, 0.5μl dNTPs, 1μl of polymerase. The reaction mixture was then subjected to 35 cycles of denaturation at 94°C for 1min, annealing at 55°C for 1min, and extension at 72°C for 1min. The products were then visualized for 511bp by ethidium bromide agarose gel staining [30]. Amplified products were then extracted from the gels using Qiaquick Gel extraction kit (QIAGEN, INC, Valencia, Calif) and both strands were sequenced by using a Big Dye Terminator Cycle Sequencing kit (Applied Biosystems). The CprM sequences were confirmed by BLAST (www.ncbi.nlm.nih.gov/BLAST). The forward and reverse sequences were aligned and manually edited using Codon Code aligner v.7.0.1 software to obtain the consensus sequence. New partial CprM sequences were submitted to GenBank at www.ncbi.nlm.nih.gov/genbank (accession number MG053110-MG053173).

### Complete genome sequencing

Full genome sequencing of viral genomes was done using Ion Proton system (Life technologies, USA). Briefly, products were purified, size selected, amplified and quantified. Clonal amplification was carried out by emulsion PCR and the Ion sphere particles were deposited on to Ion PI chip. All proton quality-approved, trimmed and filtered (against human genome) data were exported as BAM files for bioinformatics analysis. Unmapped reads were quality filtered with mean quality score > = 20, minimum length 20 and trimmed using PrinSeq-Lite program. Resulting high quality reads were assembled using MIRA v4.0.2 assembler and contigs were annotated using BLAST against NCBI database. Complete genome sequences were submitted to GenBank at www.ncbi.nlm.nih.gov (accession number MG272272-MG272274).

### Phylogenetic analysis

The sequences obtained in the present study and other sequences retrieved from GenBank were aligned using MAFFT online alignment tool [31]. Phylogenetic trees were constructed using Maximum Likelihood method based on Tamura Nei model in MEGA 6.06 software [32]. Genetic distances were calculated using the p-distance model of nucleotide and amino acid substitution. The robustness of the resulting tree was assessed with 1000 bootstrap replicates.

## Results

### Patient characteristics

During 2016-dengue season, serum samples from 109 NS1 positive patients were subjected to RT-PCR and 53(48.6%) scored positive for DENV-RNA. Further, 11 cell culture-grown DENV isolates obtained from additional NS1 positive patients were subjected to RT-PCR. Of the 64 patients, age of the patients ranged from 5 months to 65 years with median age of 28.6 years. Male (n = 35) to female (n = 29) ratio was 1: 0.8. Based on WHO 2009 guidelines, 63patients were categorized as dengue illness of which 59 without warning signs and 4 with warning signs. One patient was classified as severe dengue. Details are provided in Table 1.

**Table 1:** Demographics and clinical parameters of patients infected with DENV with different serotypes.

Sr. No.	Sample No.	Sample type	NS1 Detection	Age	Gender	Clinical Manifestation	GenBank Accession No.
**Dengue Serotype 1 and genotype V**							
1	S44	Serum	pos	36	M	DwoWS	MG053110
2	S58	Serum	pos	32	F	DwoWS	MG053111
3	S59	Serum	pos	18	M	DwoWS	MG053112
4	S105	Vero isolates	pos	27	F	DwoWS	MG053113
5	S5	Serum	pos	10	F	DwoWS	MG053114
6	S19	Serum	pos	24	M	DwoWS	MG053115
7	S16	Serum	pos	10	M	DwoWS	MG053116
8	S10	Serum	pos	4	M	DwoWS	MG053117
9	S51	Serum	pos	47	M	DwoWS	MG053118
**Dengue Serotype 2 and genotype IV (Cosmopolitan)**							
10	141	Serum	pos	30	M	DwoWS	MG053119
11	812	Serum	pos	26	F	DwoWS	MG053120
12	815	Serum	pos	22	M	DwoWS	MG053121
13	892	Serum	pos	60	F	DwoWS	MG053122
14	1008	Serum	neg	21	F	DwoWS	MG053123
15	1053	Serum	pos	20	M	DwoWS	MG053124
16	1571	Vero isolates	pos	25	F	DwoWS	MG053125
17	S107	Vero isolates	pos	16	M	DwoWS	MG053126
18	S47	Serum	pos	25	M	DwoWS	MG053127
19	S57	Serum	pos	24	M	DwoWS	MG053128
20	S77	Serum	pos	5 month	M	DWS	MG053129
21	S85	Serum	pos	11	F	DWS	MG053130
22	S94	Serum	pos	27	M	DwoWS	MG053131
23	S87	Serum	pos	35	M	DwoWS	MG053132
24	S25	Serum	pos	25	F	DwoWS	MG053133
25	S26	Serum	pos	26	M	DwoWS	MG053134
26	S52	Serum	pos	34	F	DwoWS	MG053135
27	S53	Serum	pos	50	M	DwoWS	MG053136
28	S66	Vero isolates	pos	41	M	DwoWS	MG053137
29	S67	Vero isolates	pos	38	F	DwoWS	MG053138
30	S97	Vero isolates	pos	22	M	DwoWS	MG053139
31	S100	Vero isolates	pos	18	M	DwoWS	MG053140
32	S4	Serum	pos	16	F	DwoWS	MG053141
33	S15	Serum	pos	12	F	DwoWS	MG053142
34	S9	Serum	pos	34	F	DwoWS	MG053143
35	S78	Serum	neg	21	F	DwoWS	MG053144
**Dengue Serotype 3 and genotype III**							
36	1389	Serum	pos	19	F	DwoWS	MG053145
37	984	Serum	pos	18	F	DwoWS	MG053146
38	S108	Serum	pos	24	M	DwoWS	MG053147
39	S73	Serum	pos	41	F	DwoWS	MG053148
40	S45	Vero isolates	pos	26	F	DwoWS	MG053149
41	S33	Serum	pos	25	F	DwoWS	MG053150
42	S111	Vero isolates	pos	30	F	DwoWS	MG053151
43	S56	Serum	neg	42	M	DwoWS	MG053152
44	S54	Serum	pos	39	M	DwoWS	MG053153
45	S50	Vero isolates	pos	13	M	DwoWS	MG053154
46	S74	Serum	pos	20	M	DwoWS	MG053155
47	S112	Vero isolates	pos	20	M	DwoWS	MG053156
48	S1	Serum	pos	26	F	DwoWS	MG053157
49	S76	Vero isolates	pos	8	M	DwoWS	MG053158
50	S81	Serum	pos	10	M	DwoWS	MG053159
51	S82	Serum	pos	41	M	DWS	MG053160
52	S65	Serum	pos	18	F	DwoWS	MG053161
**Dengue Serotype 4 and genotype I**							
53	S28	Serum	pos	55	M	DwoWS	MG053162
54	S30	Serum	pos	42	M	DwoWS	MG053163
55	S46	Serum	pos	37	M	DWS	MG053164
56	S80	Serum	pos	21	M	DwoWS	MG053165
57	S2	Serum	pos	16	F	DwoWS	MG053166
58	36	Serum	pos	6	F	SD	MG053167
59	294	Serum	pos	65	F	DwoWS	MG053168
60	1018	Serum	pos	2	M	DwoWS	MG053169
61	1021	Serum	pos	20	F	DwoWS	MG053170
62	1028	Serum	pos	17	M	DwoWS	MG053171
63	S49	Serum	pos	47	F	DwoWS	MG053172
64	S41	Serum	pos	23	F	DwoWS	MG053173

DwoWS: Dengue illness without warning signs

DWS: Dengue illness with warning signs

SD: Severe dengue

### DENV serotype distribution

**Fig 1:**
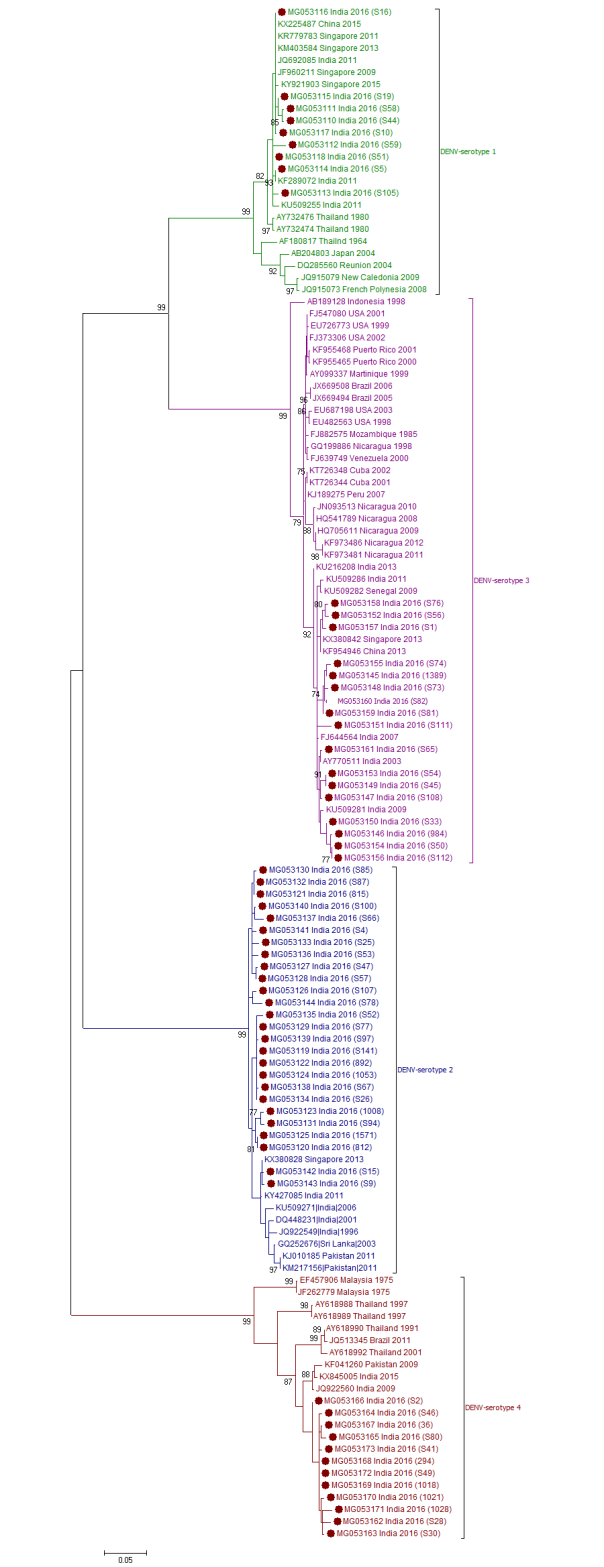
Phylogenetic analyses of CprM gene sequences from 64 DENV positive cases for serotype determination. Each strain is identified by Genbank accession number followed by country and year of isolation. Numbers at the nodes are support values for the major branches (bootstrap; 1000 replicates). The sequences obtained in this study are marked in filled colored circles. Scale bar indicates number of base substitutions per site.

Fig 1 depicts CprM gene phylogeny-based serotyping of 64 DENV sequences obtained during this study. Clearly, all the four serotypes were circulating in Pune during the 2016 season. Of these, DENV-1 was detected in 9 (14.1%, Pune-2016-DENV1), DENV-2 in 26 (40.6%, Pune-2016-DENV2), DENV-3 in 17 (26.6%, Pune-2016-DENV3) and DENV-4 in 12 (18.7%, Pune-2016-DENV4) samples. Thus, DENV-2 was found to be the predominant serotype and a substantial proportion of patients were infected with other serotypes as well. As far as serotypic distribution among different clinical forms is considered, the only severe dengue patient was infected with serotype4, patients with dengue illness without warning signs were infected with either of 4 serotypes and those with warning signs were infected with serotypes 2, 3 or 4.

### DENV genotype distribution

**Fig 2:**
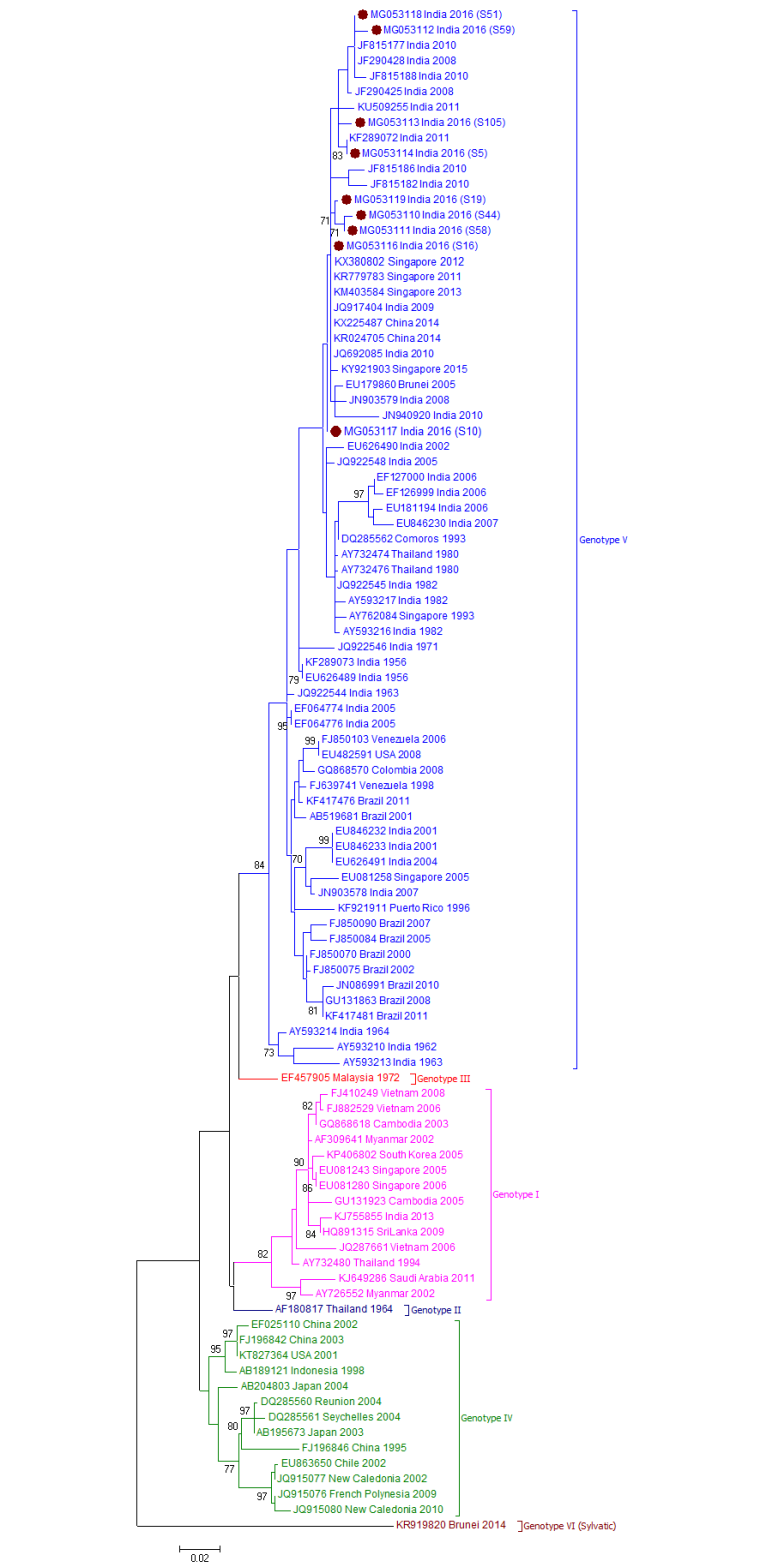
Genotyping analyses of CprM gene sequences of DENV-1 serotype isolates (n = 9) from Pune. Each strain is indicated by Genbank accession number followed by country and year of isolation. Numbers at the nodes are support values for the major branches (bootstrap; 1000 replicates). The sequences obtained in this study are marked in filled colored circles. Scale bar indicates number of base substitutions per site.

**Fig 3:**
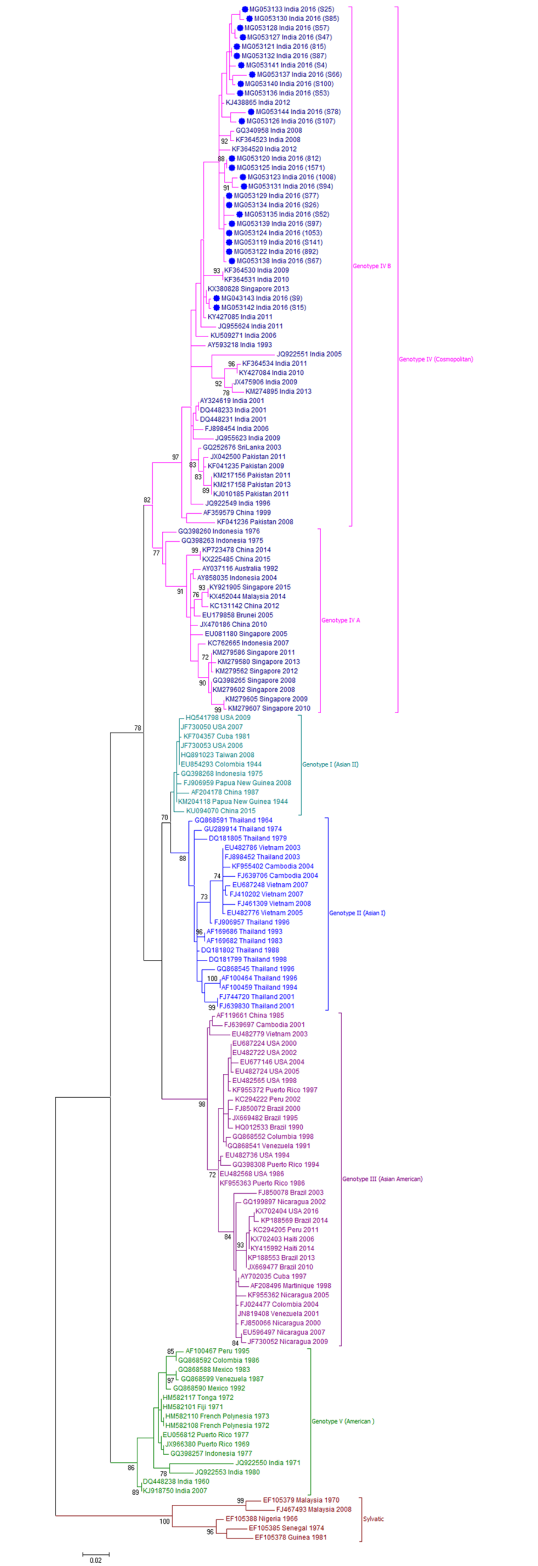
Genotyping analyses of CprM gene sequences of DENV-2 serotype isolates (n = 26) from Pune. Each strain is indicated by Genbank accession number followed by country and year of isolation. Numbers at the nodes are support values for the major branches (bootstrap; 1000 replicates). The sequences obtained in this study are marked in filled colored circles. Scale bar indicates number of base substitutions per site.

**Fig 4:**
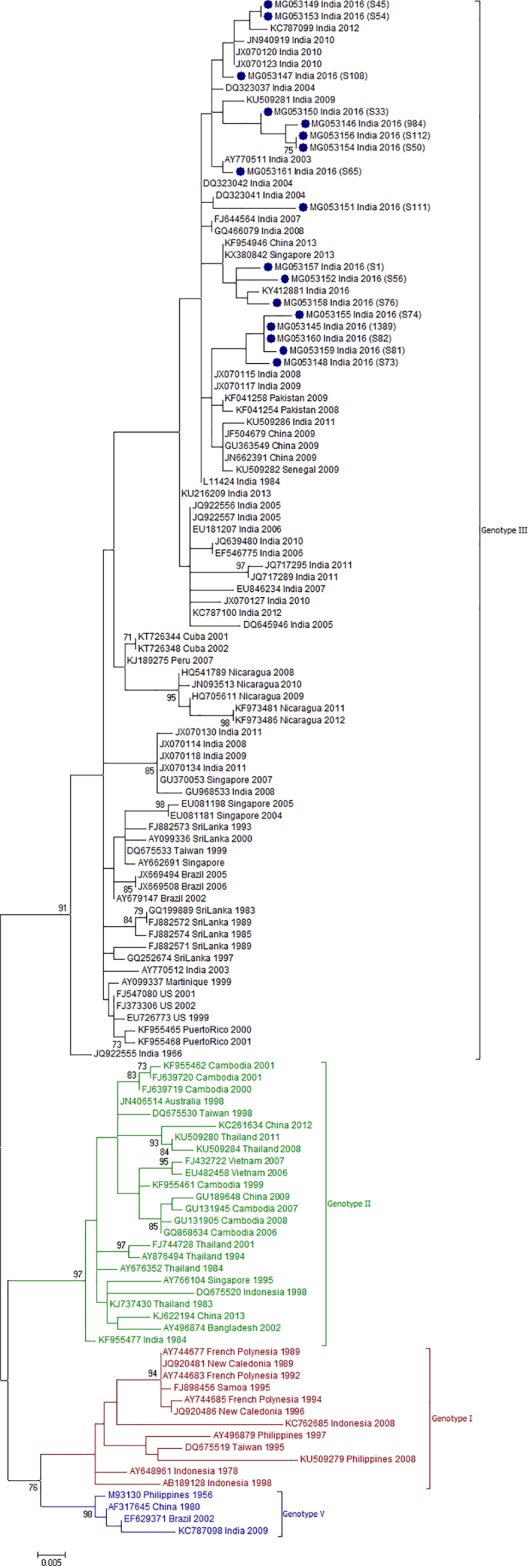
Genotyping analyses of CprM gene sequences of DENV-3 serotype isolates (n = 17) from Pune. Each strain is indicated by Genbank accession number followed by country and year of isolation. Numbers at the nodes are support values for the major branches (bootstrap; 1000 replicates). The sequences obtained in this study are marked in filled colored circles. Scale bar indicates number of base substitutions per site.

**Fig 5:**
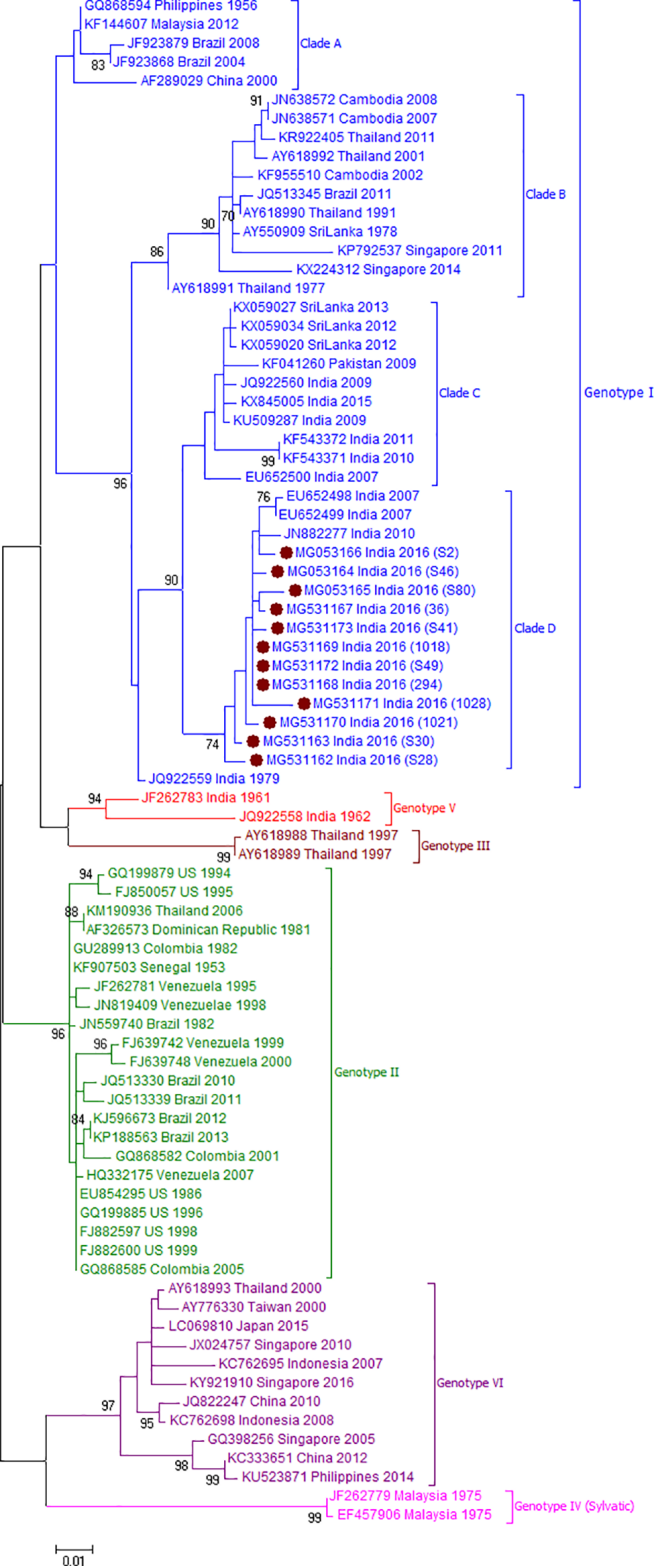
Genotyping analyses of CprM gene sequences of DENV-4 serotype isolates (n = 12) from Pune. Each strain is indicated by Genbank accession number followed by country and year of isolation. Numbers at the nodes are support values for the major branches (bootstrap; 1000 replicates). The sequences obtained in this study are marked in filled colored circles. Scale bar indicates number of base substitutions per site.

To determine the genotype distribution of DENV within each serotype, CprM gene sequences obtained during this study and sequences from different geographical locations across the globe were retrieved from NCBI database and used for phylogenetic analyses (Figs 2–5).

For DENV-1 strains, phylogenetic tree revealed clustering of DENV-1 sequences into six genotypes. The Pune-2016-DENV1isolates (n = 9) grouped into American/African (AM/AF) or genotype V together with other Indian isolates from 1962 to 2011(Fig 2). As reported earlier (Cecilia et al, 2017), one isolate from Kerala, 2013 (KJ755855) belonged to Asian or genotype I. The Pune-2016-DENV1 sequences were similar with 99.8 ± 0.3% nucleotide identities. The current sequences clustered with isolates from India (2008–2016), Singapore (2011–15), China (2014) and Brunei (2005).

As evident from Fig 3, DENV-2 sequences were classified into six genotypes. Pune-2016 sequences (n = 26) grouped together in Cosmopolitan or genotype IVand exhibited 99.3 ± 0.3% nucleotide similarity. This genotype is divided into two geographically distinct lineages, lineage A (isolates from Southeast Asia, China and Oceania) and lineage B (isolates mostly from Indian subcontinent). Pune-2016 sequences belonged to lineage B and clustered with strains from India (2008–12), Pakistan (2008–13), China (1999), Singapore (2013) and Sri Lanka (2003).

Phylogenetic analysis classified Pune-2016-DENV3 (n = 17) CprM sequences in genotype III (Fig 4) with nucleotide sequence similarity of 99.3 ± 0.3%. Genotype III strains exhibit wide geographic distribution from Asia, Caribbean, Americas and Europe.Pune-2016 sequences were closely related to the other isolates from India (2004–2016), China (2009, 2013), Singapore (2009), Pakistan (2008–09) and a single isolate from Senegal (2009) with 99.4 ± 0.3% nucleotide similarities. Among the other India isolates, 1984-isolate (KF955477) grouped into Genotype II while a single isolate from northern India (KC787098, 2009) was assigned to genotype IV and was found to be closely related to DENV-3 prototype strain of Philippines, 1956 (M93130).

DENV-4, the rare serotype in India was previously reported in 2003 from Delhi, and 2007 from Hyderabad and 2010 from Kerala [33–35]. In Maharashtra state, last report of DENV-4 cases was in 1975 from Amalner district and later detected in Pune in 2009 after a gap of 30 years [36]. Phylogenetic analysis revealed that DENV-4 sequences have been grouped into 5 genotypes with an inter-genotypic sequence divergence of > 6% [10–12, 37, 38]. Our data documented that (1) the Pune-2016 viruses (n = 12) formed a distinct cluster within genotype I and (2) 11 sequences earlier classified elsewhere [39, 40] as genotype II constituted a separate cluster (Fig 5) that included isolates mostly from East and Southeast Asian countries such as Japan, China, Taiwan, Indonesia, Singapore and Philippines.

We further compared percent nucleotide divergence in CprM region among different clusters within genotype I and different clusters constituting genotypes within serotype IV (Table 2). The novel cluster including current Pune strains was 3.0±0.6% to 5.6±0.8% divergent when compared to the other clusters/clades within genotype I and tentatively designated as clade D. DENV-4 viruses isolated in 2007 (EU652498-EU652499) and 2010 (JN882277) from two southern Indian states together with Pune-2016 sequences belonged to clade D. The distinct cluster of sequences earlier classified as genotype II was 6.8% - 10.2% different from the known genotypes I–V (Table 2). These results suggested that this cluster may represent a novel genotype VI.

**Table 2:** Nucleotide diversity in CprM region between (A) clades within genotype I and (B) across genotypes of DENV-4 viruses.

**(A)**	Clade A	Clade B	Clade C		
Clade A	--	--	--		
Clade B	5.5 ± 0.9	--	--		
Clade C	5.0 ± 0.9	5.8 ± 0.9	--		
**Clade D**	**4.6 ± 0.9**	**5.6 ± 0.8**	**3.0 ± 0.6**		
**(B)**	GT* I	GT II	GT III	GT IV (sylvatic)	GT V
GT I	--	--	--	--	--
GT II	6.9±0.9	--	--	--	--
GT III	7.8±1.0	7.3±1.0	--	--	--
GT IV (sylvatic)	10.9±1.2	9.3±1.3	10.7±1.3	--	--
GT V	7.4±0.9	6.2±0.9	7.3±1.0	11.5±1.2	--
**GT VI**	**8.6±1.0**	**6.8±1.0**	**8.9±1.1**	**10.2±1.2**	**8.0±0.9**

* GT—abbreviation for genotype

--indicates blank spaces

**Fig 6:**
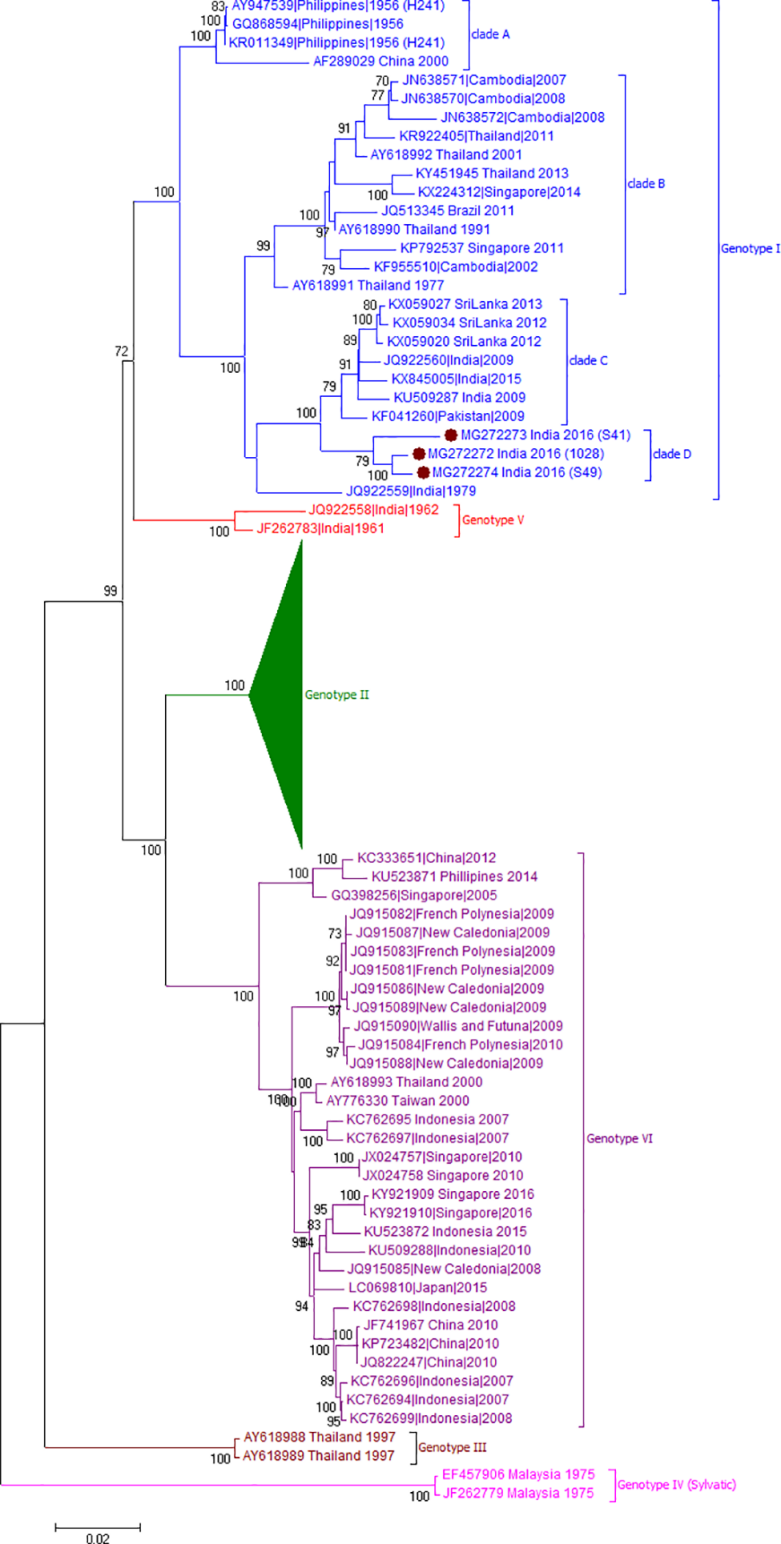
Genotyping analyses of complete gene sequences of DENV-4 serotype isolates (n = 3) from Pune. Each strain is indicated by Genbank accession number followed by country and year of isolation. 141 Genotype II sequences obtained from Genbank are shown here as compressed tree (S1 Text provides the accession numbers). Numbers at the nodes are support values for the major branches (bootstrap; 1000 replicates). The sequences obtained in this study are marked in filled colored circles. Scale bar indicates number of base substitutions per site.

For confirmation of these observations, full genome sequence analysis was done. We obtained complete genome sequences of three DENV-4 strains, isolated in Vero cells (accession number MG272272-MG272274). The complete genome lengths of Pune-2016 isolates are 10653 nucleotides (nt). The length of 5’ and 3’ untranslated regions are 103 nt and 386 nt respectively with an ORF of 10164 nt coding for 3388 amino acids. As evident from Fig 6, phylogenetic analysis on complete genome sequences confirmed emergence of a novel clade “D” within genotype I that differed by 3.3 to 5.9% (nt) and 1.2 to 1.9% (aa) from the other clades (Table 3). Pune-2016 complete genome sequences showed nucleotide similarity of 99.2 ± 0.1% among themselves and diversity of 3.3 ± 0.1% when compared with Pune, 2009 isolate (JQ922560). Genomic diversity within genotype I was highest of 4.4% as compared to other genotypes of DENV-4 viruses. The existence of an additional genotype VI was confirmed by the full genome analysis with nucleotide divergence of 6.7% - 13.5% across genotypes I to V (Table 3). Inter-genotypic divergence for genotype I to VI ranged from 6.7 to 13.7% in nucleotide and 2.2 to 5.2% in amino acid sequences (Table 3).

**Table 3:** Nucleotide and amino acid diversity in complete genome between (A) clades within genotype I and (B) across genotypes of DENV-4 viruses. Pairwise distances and standard errors of nucleotide and amino acid diversity are displayed in lower-left and upper-right matrix respectively.

**(A)**	Clade A	Clade B	Clade C	**Clade D**		
Clade A	--	1.9±0.2	1.8±0.2	**1.9±0.2**		
Clade B	5.4±0.2	--	1.7±0.2	**1.7±0.2**		
Clade C	5.3±0.2	5.4±0.2	--	**1.2±0.2**		
Clade D	**5.7±0.2**	**5.9±0.1**	**3.3±0.1**	--		
**(B)**	GT* I	GT II	GT III	GT IV (sylvatic)	GT V	**GT VI**
GT I	--	2.7±0.2	2.9±0.3	4.8±0.4	3.0±0.2	**3.1±0.3**
GT II	7.9±0.2	--	2.8±0.3	4.5±0.4	3.0±0.3	**2.2±0.2**
GT III	9.2±0.2	9.2±0.3	--	4.4±0.4	3.2±0.3	**3.1±0.3**
GT IV (sylvatic)	13.4±0.3	13.3±0.3	13.7±0.3	--	5.2±0.4	**4.7±0.4**
GT V	7.3±0.2	7.1±0.2	8.5±0.3	13.4±0.3	--	**3.2±0.2**
**GT VI**	**8.2±0.2**	**6.7±0.2**	**9.4±0.3**	**13.5±0.3**	**7.6±0.2**	--

* GT—abbreviation for genotype

--indicates blank spaces

To understand the mutation sites associated with the divergence of Pune-2016 DENV-4 isolates to “clade D”, amino acid sequence comparison with different clades of genotype I and Indian isolates of genotype V was carried out (Table 4). Unique substitutions in coding region of clade D was identified in comparison to reference strain H241 isolated in Philippines, 1956 (AY947539, clade A).A total of 7 amino acid changes in polyprotein (M271I, I411V, K479T, N645S, F945L, V1262Aand C1310R) were found to be specific to clade D. In fact, none of the other genotype I clades exhibited these amino acid substitutions. Individual protein analysis revealed that amino acid substitutions specific to Clade D were confined to membrane glycoprotein precursor (n = 1), envelope (n = 3), NS1 (n = 1), and NS2A (n = 2) regions (Table 4). Envelope region showed two and one amino acid substitutions in domain II (I132V, K200T) and domain III (N366S) respectively. Domain III (residues 300–495) is responsible for receptor binding and contains virus neutralizing epitopes. Two amino acid substitutions in NS2A region (V136A and C184R) might affect virus replication. As compared to the reference strain, clades C and D shared identical substitutions at only one amino acid position in NS3 (M605V) region. Three amino acid substitutions specific to clade C at positions 130, 202 in envelope and at position 383 in NS3wasreversed back to the original amino acids of reference strain in clade D.

**Table 4:** Comparative analyses of amino acid substitutions among 4 clades of genotype I, genotype V and Indian isolate, 1979 to corresponding residues in the reference strain, H241 (AY947539, Philippines 1956) of clade A.

Sr No.	Genomic region	Polyprotein position	Gene position	Reference strain (H241)	Genotype I				JQ922559(India, 1979)	Genotype V(India, 1961–62)
					Clade A	Clade B	Clade C	Clade D(Pune 2016)		
1	prM (114–279,166aa)	269	156	V	*	I	I	I	I	*
2		**271**	**158**	**M**	*	*	*	**I**	*	*
3	Env (280–774, 495aa)	*409*	*130*	*V*	*	*	*I*	*	*	*
4		**411**	**132**	**I**	*	*	*	**V**	*	*
5		**479**	**200**	**K**	*	*	*	**T**	*	*
6		*481*	*202*	*K*	*	*	*N*	*	*	*
7		512	233	Y	*	H	H	H	*	*
8		**645**	**366**	**N**	*	*	*	**S**	*	*
9	NS1 (775–1126, 352aa)	776	2	T	*	M	M	M	M	M
10		903	129	K	*	R	R	R	R	R
11		**945**	**171**	**F**	*	*	*	**L**	*	*
12	NS2A (1127–1344, 218aa)	**1219**	**93**	**R**	*	*	*	**K!**	*	*
13		**1262**	**136**	**V**	*	*	*	**A**	*	*
14		1281	155	R	*	K	K	K	K	*
15		**1310**	**184**	**C**	*	*	*	**R**	*	*
16	NS2B (1345–1474, 130aa)	1433	89	I	*	V	V	V	V	V
17	NS3(1475–2092, 618aa)	1536	62	T	*	S	S	S	S	*
18		1645	171	T	*	I	I	I	I	I
19		1795	321	A	*	T	T	T	T	*
20		*1857*	*383*	*I*	*	*	*V*	*	*	*
21		1954	480	K	*	R	R	R	R	*
22		2079	605	M	*	*	V	V	*	*
23	NS4A (2093–2219, 127aa)	2209	117	V	*	*	A	A!!	*	A
24	2K peptide (2220–2242, 23aa)	2240	21	I	*	V	V	V	V	*
25	NS4B(2243–2487, 245aa)	2440	198	V	*	I	I	I	I	I
26	NS5(2488–3387, 900aa)	2733	246	R	*	*	*	K	K	*
27		2734	247	H	*	*	Y∉	Y	*	*
28		2743	256	V	*	A	A	A	A	*
29		2762	275	T	*	*	A∉	A	*	*

*—sequence similar to reference strain Philippines, 1956

**Bold**–amino acid substitution unique to clade D

**!**—amino acid substitution unique to clade D except MG272272 (1028)

!! - amino acid substitution unique to clade D except MG272273 (S41)

**Italics**—amino acid substitution unique to clade C

∉—amino acid substitution unique to clade C except KU509287

## Discussion

This study documents co-circulation of all the four serotypes of DENV during a single season at Pune, India. Interestingly, though DENV-2 was the most prevalent serotype (40.6%), 18.7% isolates belonged to serotype IV. This is especially important since this serotype was introduced in this city in 2009 after a gap of 30 years [36]. Since 2005, DENV-1, 2 and 3 were shown to co-circulate. However, each year was dominated by a single serotype; DENV-1 in 2005 and 2007, DENV-2 in 2008 and DENV-3 in 2009. In 2010, both DENV-2 and DENV-3 were co-dominant [41]. 2016 witnessed prevalence of all the four serotypes to an appreciable extent and presents possible risk of secondary infection with serotype 4 leading probably to severe disease. Though one patient infected with serotype 4 led to severe disease, no conclusions can be made because of small numbers.

On account of highest mutation rate among the Flavivirus group, DENV serotypes are divided into different genotypes and further into lineages or clades [10–12]. Infection of populations with a new genotype not exposed to earlier or with a virus with lineage shift within genotype has been attributed to severe form of disease [19, 42–44]. In the light of these observations, it is important to note that Pune-2016 DENV-4 isolates formed a distinct clade-D within genotype I that were 3.0%- 5.6% (CprM) and 3.3%-5.9% (complete genome) divergent from clades A, B and C. Interestingly, viruses isolated earlier (2007, 2010) from two southern states also belonged to this clade. Identification of novel clade within genotype I emphasizes high rate of genomic diversity in this continually evolving genotype of DENV-4 viruses. It would be desirable to assess the role of this clade in disease severity when presenting with primary or secondary infection. Further monitoring is essential to identify emergence of novel DENV-4 viruses, especially, as introduction of dengue vaccine remains a distinct possibility in endemic areas including India. As far as serotypes I, II and III are concerned, similar to earlier reports from India [25, 45–48], persistent circulation of genotype V of DENV-1, genotype IV of DENV-2 and genotype III of DENV-3 was noted.

Another significant observation of this study is the identification of an additional genotype (VI) within serotype-4. This genotype includes viruses from East Asia, Southeast Asia and Oceania countries, isolated during 2000-2016that were earlier grouped in genotype II [39–40, 49–50]. Genotype VI is proposed since > 6% divergence from other genotypes was observed as evidenced by complete genome based phylogenetic analysis (Table 3, Fig 6). Further study is required to correlate disease profile of patients infected with different genotypes of DENV-4 viruses. Different regions of dengue genome like Envelope, E-NS1 and C-prM have been largely utilized for genotyping. CprM gene based genotyping is faster and economical due to usage of single set of primer pair for both amplification and sequencing [30, 51]. Our study emphasizes the utility of this region for genotyping as the CprM based observations of emergence of a new clade in genotype I or a distinct cluster within genotype II were confirmed by complete genome based analysis.

Chances of co-infection with more than one serotype are likely to be much higher when multiple dengue serotypes co-circulate in a population. Co-infection with multiple serotypes poses risk of emergence of recombinant virus strains that could have distinct properties. Co-circulation of all the four serotypes in a single outbreak has been reported earlier with 42.9% and 45.4% cases of co-infection in Karnataka and Hyderabad respectively [28, 52]. Significant co-infection (15% -43%) has been reported from northern [24, 26], and eastern India[25] without detecting all the 4 serotypes. These regions with circulation of more than one serotype simultaneously are of high significance as they are more prone to severe dengue infection [28, 52]. However, we did not find evidence of co-infection among the patients studied.

In summary, in contrast to the predominance of a single serotype observed earlier, we provide recent evidence of significant co-circulation of all the four serotypes in Pune and emergence of a novel clade in genotype I of DENV-4 viruses. In view of the role of novel strains in increased severity and vaccine availability in near future, a comprehensive molecular surveillance programme for DENV is urgently needed.

## Supporting information

S1 Text Details of accession numbers for DENV-4 genotype II sequences (n = 141) shown as compressed tree in Fig 6. (DOCX)
