# Supplementary material for: Co-circulation of all the four dengue virus serotypes and detection of a novel clade of DENV-4 (genotype I) virus in Pune, India during 2016 season
Source: PLoS One. 2018 Feb 22;13(2):e0192672. doi: 10.1371/journal.pone.0192672 (PMC5823370; doi:10.1371/journal.pone.0192672)
Supplement: S1 Text — (DOCX) [file pone.0192672.s001.docx]

**S1 Text: Details of accession numbers for DENV - 4 genotype II sequences (n=141) shown as compressed tree in Figure 6.**

AF326573, KM190936, GU289913, JN559740, KF907503, FJ226067, FJ810417, GQ199879, GQ199880, GQ252675, FJ850057, JF262782, KJ160504, FJ024476, JF262781, EU854295, FJ882600, EU854297, FJ850058, GQ199885, FJ882598, FJ882597, GQ199878, GQ199881, GQ199883, GQ199884, GQ199882, FJ882595, FJ882596, FJ024424, FJ882599, FJ882601, EU854296, FJ850059, FJ639736, FJ639744, FJ639739, FJ639742, FJ639742, FJ639745, FJ639748, FJ639738, FJ639764, FJ639737, JQ513334, JQ513335, JQ513336, JQ513337, EU854299, GQ868645, FJ882583, FJ882590, FJ882591, GQ868643, FJ882589, GQ868642, GQ868644, GQ868585, FJ882586, FJ882587, HQ332175, FJ182016, FJ882580, FJ882584, JN819406, FJ182017, HQ332172, HQ332173, HQ332176, FJ882585, EU854301 ,FJ882592 ,HQ332174 ,GQ199876, EU854300, FJ882581, FJ882582, FJ882588, FJ639773, FJ850095, JQ513330, JQ513330, JQ513331, JQ513332, JQ513333, JQ513341, JQ513340, GQ868583, GQ868584, JN559741, JN983813, KJ579241, KJ579242, KJ579243, KJ596665, KJ596658, KJ579246, KJ596663, KJ596668, KJ596659, KJ579240, KJ579244, KJ596670, KJ596674, KJ596662, KJ596666, KJ579245, KJ596664, KJ596667, KJ596673, KJ596672, KJ596669, KJ579248, KJ579247, KJ596671, KJ596660, KU513441, KT794007, KP188562, KP188566, KP188563, KJ596661, KP188558, KP188560, KP188559, KP188561, KP188564, KP188557, JQ513338, JQ513339, JQ513342, JQ513343, JQ513344, GQ868579, GQ868580, GQ868581, GQ868582, JN819409, KP188565, KP140942, KT276273
